# A modifiable factors-based model for detecting inactive individuals: are the European assessment tools fit for purpose?

**DOI:** 10.1093/eurpub/ckac116

**Published:** 2022-09-09

**Authors:** X Mayo, E Iglesias-Soler, G Liguori, R J Copeland, I Clavel, F del Villar, A Jimenez

**Affiliations:** Observatory of Healthy & Active Living of Spain Active Foundation, Centre for Sport Studies, King Juan Carlos University, Madrid, Spain; Performance and Health Group, Department of Physical Education and Sport, Faculty of Sports Sciences and Physical Education, University of A Coruna, A Coruña, Spain; University of Rhode Island, Kingston, RI, USA; Advanced Wellbeing Research Centre, College of Health, Wellbeing and Life Sciences, Sheffield Hallam University, Sheffield, UK; The National Centre for Sport and Exercise Medicine, Sheffield, UK; Performance and Health Group, Department of Physical Education and Sport, Faculty of Sports Sciences and Physical Education, University of A Coruna, A Coruña, Spain; Galician Sport Foundation, Galician Government, Santiago, Spain; Observatory of Healthy & Active Living of Spain Active Foundation, Centre for Sport Studies, King Juan Carlos University, Madrid, Spain; Observatory of Healthy & Active Living of Spain Active Foundation, Centre for Sport Studies, King Juan Carlos University, Madrid, Spain; GO fit LAB, Ingesport, Madrid, Spain

## Abstract

**Background:**

The lack of systematic factors affecting physical inactivity (PIA) challenges policymakers to implement evidence-based solutions at a population level. The study utilizes the Eurobarometer to analyse PIA-modifiable variables.

**Methods:**

Special Eurobarometer 412 physical activity (PA) data were analysed (*n* = 18 336), including 40 variables along with the International PA Questionnaire. PIA was used as the dependent variable. Variables considered were alternatives to car, places, reasons and barriers to engaging in PA, memberships to clubs and categorical responses about the agreement extent with the area, provision of activities and local governance statements. Logistic regression was used to identify variables contributing to PIA. Beta values (*β*), standard errors, 95% confidence intervals, the exponentiation for odds ratio and Cox & Snell and Nagelkerke *R*^2^ were indicated.

**Results:**

The resulting model correctly identified 10.7% inactives and 96.9% of actives (*R*^2^ of Nagelkerke: 0.153). Variables contributing to the detection of PIA were (*P* ≤ 0.01): having a disability or an illness, not having friends to do sport with, lacking motivation or interest in and being afraid of injury risk. Additionally, totally agreeing, tend to agree and tend to disagree regarding the extent of local providers offering enough opportunities to be more active also contributed to the model.

**Conclusions:**

The model reported a limited ability to detect modifiable factors affecting PIA, identifying a small percentage of inactive individuals correctly. New questions focused on understanding inactive behaviour are needed to support the European PA public health agenda.

## Introduction

Past epidemiological studies over the years have identified that physical inactivity (PIA), i.e. not achieving current guidelines for physical activity (PA), is a risk factor for developing chronic diseases and increasing premature mortality.^1^ Nevertheless, reviews analysing causal variables fail to find systematic factors influencing PIA.^2^^,^^3^ For tackling this behaviour, holistic solutions able to translate scientific evidence on concrete policy actions should be a primary policy priority.^4^^,^^5^ In this sense, understanding how different variables influence PIA is of great importance to designing programmes aimed at reducing this behaviour.^2^^,^^3^ Thereby, programmes affecting several of these variables may be more relevant to creating population level-changes compared to an intervention or policy affecting only one variable.^4^^,^^5^

Studies attempting to build intervention models intended to increase policy effectiveness around PIA are infrequently assessed.^6^^,^^7^ Often, these studies use regression models to understand active behaviour, as opposed to inactivity, and with small samples and a limited number of questions to reasonably explain the behaviour.^6^^,^^7^ Further, the dependent variable of these studies rarely categorises behaviour via a dichotomous outcome in a sort of PA recommendations (i.e. active vs. inactive), limiting the applications in policy development.^1^

Considering all this, the ‘Sport and Physical Activity’ Eurobarometer surveys implemented in the European Union might be a more appropriate tool to identify variables required to understand PIA.^4^ This is because ecological approaches (such as that taken as part of the Eurobarometer survey) are better on improving the prediction capacity of inactive behaviour.^8^ Theoretically, Eurobarometers might help the European Union Health and Sport Secretariats to make decisions about how best to address inactivity; however, the outcomes to date have shown limited effectiveness.^5^ Considering this limitation, research into some particular fields of human behaviour might help to identify new potential factors that could be included in new public health surveillance questionnaires, while at the same time, limiting the use of factors that do not impact PIA.^4^

*A priori* relevant domains included in the Eurobarometer have previously shown in other studies an impact on active behaviour. Some factors have shown to increase the odds of being physically active, such as (i) using alternatives to the car in forms of walking and cycling^9^; (ii) particular places more suitable for engaging in active behaviour, such as streets, roads or between places,^10^ fitness centres, park and outdoors or home^11^; (iii) concrete personal reasons to be active, such as for improving health and appearance,^7^ enjoying being physically active^6^ or for the sake of competitiveness^7^; (iv) the simple perception of barriers, which are associated with a reduced likelihood of sufficient PA participation,^6^ which includes not having infrastructure close,^10^ lack of time,^11^ interest^7^ or motivation^12^; and (v) being a member of a recreational facility vs. not.^13^

There is also growing evidence to suggest that a supportive environment can increase the odds of being physically active, including (vi) enhancing the perceived extent of opportunities to be physically active, including the availability and convenience of recreational facilities, places or spaces to be active^14^; and (vii) the awareness and dissemination of the opportunities to be active by the provision of programmes and activities of local sports clubs and other local providers.^14^ On the contrary, (viii) the effect of policy and legislation on PA participation seems to be limited,^4^ since the perception of local authorities accomplishing their responsibility of promoting active living do not increase the odds of being physically active.^14^ The challenge, therefore, is to identify how best to support policymakers to implement changes across all of these areas collectively, with lower costs per person, helping to overcome the lack of change maintenance and the elevated cost usually presented in individually oriented interventions.^15^ Therefore, developing policies to drive population change that are evidence-based is much needed.

This study aimed to utilize the Eurobarometer variables to elucidate the modifiable personal, social and environmental determinants of PA by implementing a logistic regression analysis. The Eurobarometer presents a unique opportunity to consider the variables affecting PIA, given the plethora of questions that the survey asks from different perspectives. In this sense, in regression analysis, the redundant variables are eliminated from the model, just selecting the variables that better explain the variance. Additionally, odd ratio analysis allows understanding what factors are critical in the presence of others, from the same type or another. Identifying factors that are amendable to change (as opposed to fixed factors such as age or gender) seems particularly relevant here as this will help policymakers to take multi-level evidence-informed decisions regarding intervention commissioning and policy action.

## Methods

### Data source

Data from the Special Eurobarometer 412 (Wave EB80.2; fieldwork: November–December 2013, publication: March 2014) were obtained, with an initial sample of *n* = 27 919 from the 28 European Union countries. Only respondents with all the questions answered and over the age of 18 were analysed since PA recommendations differ between age ranges.^1^ In the Eurobarometers, primary sampling units are selected with a multi-stage sampling design from each of the administrative regions in every country. This sampling unit’s selection is proportional to every country’s population size from sampling frames stratified by the urbanization’s degree.^16^

### Measures

Several dichotomic variables were analysed such as environmentally friendly alternatives to car, places, reasons and barriers (i.e. preventing reasons) to engage PA or sports, memberships to clubs and three categorical responses regarding the extent of agreement or disagreement with some statements about the area, provision and local governance regarding PA and sports.

The variables included in the analysis with their particular code for further consideration are as follows^16^: the dichotomic questions (Yes or No) were about (QA4) the regular use of environmentally friendly alternatives to using a private in the past 6 months in order taken any action to fight climate change such as walking, biking, taking public transport or car-sharing; (QD7) the places used to engage PA or a sport such as (QD7.1) a health or fitness centre, (QD7.2) a sports club, (QD7.3) a sports centre, (QD7.4) at the school or the university, (QD7.5) at work, (QD7.6) at home, (QD7.7) on the way between home and school, work or shops, (QD7.8) in a park or outdoors or (QD7.9) elsewhere; (QD8) the reasons to engage in PA or sports such as (QD8.1) improve the health, (QD8.2) improve the physical appearance, (QD8.3) to counteract the effect of ageing, (QD8.4) to have fun, (QD8.5) to relax, (QD8.6) to be with friends, (QD8.7) to make new acquaintances, (QD8.8) to meet people from other cultures, (QD8.9) to improve physical performance, (QD8.10) to improve fitness, (QD8.11) to control the weight, (QD8.12) to improve self-esteem, (QD8.13) to develop new skills, (QD8.14) for the spirit of competition and (QD8.15) to better integrate into society or (QD8.16) other; (QD9) the main barriers (i.e. reasons currently preventing) the respondent from practicing PA or sports such as (QD9.1) not having enough time, (QD9.2) being too expensive, (QD9.3) not liking competitive activities, (QD9.4) not having suitable or accessible sport infrastructure close, (QD9.5) having a disability or an illness, (QD9.6) not having friends to do sport with, (QD9.7) feeling discriminated against by other participants, (QD9.8) lacking motivation or not being interested, (QD9.9) being afraid of the risk of an injury, (QD9.10) doing already sports regularly or (QD9.11) other; (QD10) being a member of any club where practicing sport or recreational PA such as (QD10.1) a health or fitness centre, (QD10.2) a sport club, (QD10.3) a socio-cultural club that includes sport in its activities, (QD10.4) other or (QD10.5) not being a member of any club; and (BD12) volunteering supporting sporting activities. Additionally, ‘do not know’ was a possible answer for every question (i.e. QD7.10, QD8.17, QD9.12 and QD10.6).

Some categorical questions regarding the extent of agreement or disagreement with some statements (QD11) were also analysed, including: (QD11.1) the extent of opportunities to be physically active in an area, (QD11.2) the extent of local sports clubs and other local providers offering opportunities to be physically active and (QD11.3) the local authorities not doing enough for its citizens in relation to physical activities. There were four possible answers to these questions: ‘totally agree’, ‘tend to agree’, ‘tend to disagree’ and ‘totally disagree’.

To assess compliance with the World Health Organization’s PA recommendations as the dependent variable, the questions regarding the short form of the International PA Questionnaire (IPAQ) provided by the Eurobarometer were analysed. The IPAQ questionnaire measures the intensity, frequency and duration of the PA performed in the last 7 days. This information was obtained by the questions inquiring about the number of days practicing vigorous (QD3A) and moderate (QD4A) PA and walking activity (QD5A), and their respective minutes during (i.e. QD3B, QD4B and QD5B, respectively) those days. Since minutes are reported in block times, data were recalculated as previously reported for reporting minutes.^17^ An answer of ‘30 min or less’ was adjusted to 15 min, an answer of ‘31–60 min’ was modified to 45 min, ‘61–90 min’ was recoded as 75 min, ‘91–120 min’ was changed to 105 min and ‘more than 120 min’ was modified to 120 min. Data were analysed following the Guidelines for data processing and analysis of the IPAQ short form,^18^ using a spreadsheet available elsewhere.^19^ Only respondents with at least one valid intensity and duration of a particular level (i.e. both variables with a different answer than ‘don’t know’) were suitable for further analysis.

Respondents were considered physically active when performing (i) at least 3 days of vigorous-intensity activity of at least 20 min per day, (ii) at least 5 days of moderate-intensity activities and/or walking for at least 30 min per day or (iii) at least 5 days combining the intensities mentioned above achieving at least 600 metabolic equivalents (MET) per minute and week (MET-min/week). For this, vigorous and moderate-intensity and walking represent 8.0, 4.0 and 3.3 METs, respectively.^18^ Individuals were classified as physically inactive (i.e. low PA levels) when not reaching any of those thresholds.

### Statistical analysis

Logistic regression with a likelihood ratio statistic with a backward stepwise method was used to create a reliable removal criterion while reducing the possibility of making a Type II error. Nineteen steps were used in total. As previously explained, PIA was used as a dependent variable (i.e. ‘0’ in the case of not being physically inactive and ‘1’ while being physically inactive). The covariables used in the model were the previously mentioned (i.e. QA4, QD7.1–7.10, QD8.1–17, QD9.1–12, QD10.1–6, BD12, QD11.1–3). The four answers of the three categorical questions (i.e. QD11.1, QD11.2 and QD11.3) were converted into dummy variables using the deviation (first) contrast (i.e. Totally disagreeing).

Data reported were beta values (*β*) and standard errors, the exponentiation for odds ratio [Exp(*β*)] with the 95% confidence interval, and the significance level for every variable. Additionally, Cox & Snell and Nagelkerke *R*^2^ were indicated. *A priori* alpha level was set at 0.05. The statistical analysis was carried out using IBM SPSS version 19 (Armonk, NY, USA).

## Results

A final sample of *n* = 18 336 (inactive: *n* = 4757, active: *n* = 13 578) were considered. After 18 steps in the backward stepwise method, Step 19 is reported in table 1. In this table, the variables that help to construct the model for detecting inactive people are represented. The model showed a sensitivity of 10.7% (percentage of correctly identified inactive people) and a specificity of 96.9% (percentage of correctly identified active individuals), giving a global total percentage of 74.5% (χ^2^ = 2023.588; *DF* = 8; *P* < 001). The model presents an *R*^2^ of Cox & Snell of 0.104 and a *R*^2^ of Nagelkerke of 0.153.

**Table 1:** Logistic regression of a model for detecting inactive individuals based on modifiable factors (*n* = 18 336)

	β (SE)	Exp(β)	95% CI for odds ratio		*P*-value
			Lower	Upper	
Constant	−0.155 (0.082)	0.856			0.06
Environmentally friendly alternatives to car (QA4)	−0.083 (0.041)	0.920	0.849	0.997	0.041
Places (QD7)					
Health or fitness centre (QD7.1)	−0.479 (0.082)	0.741	0.633	0.728	<0.001
Sport club (QD7.2)	−0.435 (0.091)	0.647	0.542	0.773	<0.001
Sport centre (QD7.3)	−0.300 (0.081)	0.741	0.633	0.868	<0.001
At school or university (QD7.4)	−0.400 (0.136)	0.671	0.514	0.876	0.003
At work (QD7.5)	−1.159 (0.068)	0.314	0.275	0.358	<0.001
At home (QD7.6)	−0.535 (0.039)	0.586	0.542	0.633	<0.001
On the way between places (QD.7.7)	−0.209 (0.042)	0.811	0.747	0.881	<0.001
In a park or outdoors (QD7.8)	−0.382 (0.040)	0.682	0.631	0.738	<0.001
Reasons (QD8)					
Improving health (QD8.1)	−0.230 (0.040)	0.794	0.735	0.859	<0.001
Improving physical appearance (QD8.2)	−0.140 (0.050)	0.794	0.735	0.959	<0.001
Having fun (QD8.3)	−0.100 (0.046)	0.905	0.827	0.989	0.028
Relaxing (QD8.5)	−0.087 (0.040)	0.916	0.848	0.991	0.028
Improving physical performance (QD8.9)	−0.029 (0.050)	0.795	0.721	0.877	<0.001
Improving fitness (QD8.10)	−0.093 (0.040)	0.911	0.842	0.986	0.021
Improving self-esteem (QD8.12)	−0.151 (0.070)	0.860	0.750	0.986	0.031
Developing new skills (QD8.13)	−0.294 (0.106)	0.745	0.605	0.917	0.006
Better integrate into society (QD8.15)	−0.265 (0.132)	0.767	0.578	0.995	0.046
Another reason (QD8.16)	−0.409 (0.071)	0.665	0.578	0.764	<0.001
Barriers (QD9)					
No infrastructure close (QD9.4)	−0.167 (0.085)	0.846	0.716	1.000	0.05
Having a disability or an illness (QD9.5)	0.521 (0.052)	1.684	1.521	1.865	<0.001
Not having friends to do sport with (QD9.6)	0.314 (0.089)	1.369	1.150	1.629	<0.001
Lacking motivation or interest (QD9.8)	0.407 (0.045)	1.502	1.376	1.641	<0.001
Being afraid of the risk of an injury (QD9.9)	0.190 (0.073)	1.209	1.047	1.396	0.010
Doing already sports regularly (QD9.10)	−0.907 (0.068)	0.404	0.353	0.462	<0.001
Don’t know (QD9.12)	−0.316 (0.109)	0.729	0.589	0.902	0.004
Being a member (QD10)					
Being a member of a health/fitness centre (QD10.1)	−0.183 (0.087)	0.833	0.702	0.988	0.036
Being a member of a sport club (QD10.2)	−0.248 (0.078)	0.780	0.669	0.910	0.002
Being a member of a socio-cultural club (QD10.3)	−0.473 (0.108)	0.623	0.504	0.770	<0.001
Extent of opportunities in an area (QD11.1)					
Extent of opportunities: totally disagree					0.001
Extent of opportunities: totally agree	−0.297 (0.105)	0.743	0.606	0.913	0.005
Extent of opportunities: tend to agree	0.130 (0.99)	0.878	0.723	1.066	0.189
Extent of opportunities: tend to disagree	−0.017 (0.102)	0.983	0.805	1.200	0.866
Extent of local sports clubs and other local providers offering opportunities (QD11.2)					
Extent of sport providers: totally disagree					<0.001
Extent of sport providers: totally agree	0.392 (0.102)	1.480	1.211	1.809	<0.001
Extent of sport providers: tend to agree	0.433 (0.096)	1.542	1.278	1.861	<0.001
Extent of sport providers: tend to disagree	0.302 (0.082)	1.353	1.117	1.638	0.002

*β*, beta values; SE, standard error; CI, confidence intervals; Exp(*β*), exponential beta.

Some variables helped detect physically inactive individuals, such that the odds for an individual for being physically inactive were higher if they reported having a disability or an illness (QD9.5), not having friends to do sport with (QD9.6), lacking motivation or interest (QD9.8) and being afraid of the risk of an injury (QD9.9). Additionally, the odds for an individual for being physically inactive were also higher while totally agreeing, tend to agree and tend to disagree regarding the extent of local sports clubs and other local providers offering enough opportunities to be physically active (QD11.2) when comparing with the reference category ‘totally disagreeing’.

Most of the other variables helped to detect physically active individuals. Regarding place, the odds for an individual being physically inactive were lower, particularly when practicing PA or sports at work (QD7.5), but also while practicing at home (QD7.6), at health or fitness centres (QD7.1) and sport clubs (QD7.2). Concerning the reasons for practicing PA or sports, the odds for an individual being physically inactive were lower when practicing for improving fitness (QD8.10), relaxing (QD8.5) and having fun (QD8.3). Regarding membership, all possibilities are indicators of being physically active, showing lower odds for an individual being physically inactive. Regarding this, membership in a health or fitness centre was the one with a higher odds ratio (QD10.1).

Table 2 indicates variables that were eliminated from the model after Step 19, thus not contributing to the final model.

**Table 2:** Variables eliminated from the model for detecting inactive individuals based on modifiable factors after the Step 19 (*n* = 18 336)

	β	*P*-value
Places (QD7)		
Elsewhere (QD7.9)	0.665	0.415
Don’t know (QD7.10)	1.607	0.205
Reasons (QD8)		
Counteracting the effect of ageing (QD8.3)	2.499	0.114
Being with friends (QD8.6)	2.264	0.132
Making new acquaintances (QD8.7)	0.365	0.546
Meeting people from other cultures (QD8.8)	0.471	0.492
Controlling weight (QD8.11)	1.103	0.294
For the spirit of competition (QD8.14)	0.493	0.483
Don’t know (QD8.17)	0.832	0.362
Barriers (QD9)		
Not having enough time (QD9.1)	0.160	0.689
Being too expensive (QD9.2)	0.719	0.396
Not liking competitive activities (QD9.3)	1.078	0.299
Feeling discriminated against by other participants (QD9.7)	0.019	0.889
Another reason from preventing (QD9.11)	0.120	0.729
Being a member (QD10)		
Being a member: Other membership (QD10.4)	0.064	0.800
Not being a member (QD10.5)	0.173	0.678
Being a member: Don’t know (QD10.6)	0.145	0.704
Local authorities not doing enough for its citizens in relation to physical activities (QD11.3)		
Local authority not doing enough: totally agree	0.964	0.326
Local authority not doing enough: tend to agree	0.085	0.771
Local authority not doing enough: tend to disagree	0.051	0.821

β, beta values.

## Discussion

The main findings of the present study are that the model showed a limited ability to detect modifiable factors affecting PIA since a very small part of the variance was explained after Step 19. In this regard, the model was only able to identify a minimal percentage of inactive individuals correctly, yet detecting active participants was much more successful. Accordingly, just a few variables showed higher odds for being physically inactive: having a disability or an illness, not having friends to do sport with, lacking motivation or interest, being afraid of the risk of an injury, and totally agreeing, tend to agree and tend to disagree regarding the extent of local sports clubs and other local providers offering enough opportunities to be physically active vs. totally disagreeing.

Our study showed a limited explained variance (*R*^2^ of Nagelkerke = 0.153). Considering different dimensions was thought to inform the model better, but the total variance explained is comparable to previous regression analyses.^6^^,^^20–23^ Additionally, the fact of not including non-modifiable factors also slightly reduced the variance, since variables such as sex or age, also explain the model.^23^^,^^24^ Nonetheless, although the model detected almost all the physically active individuals, it was much less successful at identifying inactive individuals. Thus, our findings indicate this is a good survey for tracking PA behaviour but limited for helping to understand PIA. Although the first is relevant from policy development, the latter has its own importance, as PIA burdens the public health systems from both a societal and economic perspective.^25^

A results overview show that some groups of questions are directly related with reducing the odds of being inactive, such as alternatives to private car, places and reasons to be physically active, being a member and agreeing with certain statements. Only the group of barriers was related with increased odds of being inactive.

The question about environmentally friendly alternatives to using a private car showed a significant, albeit small, increased the odds of being physically active, as was previously reported.^26^ The literature indicates that when alternatives to car use are guaranteed,^27^^,^^28^ between 15% and 30% of the total PA is performed when commuting.^29^^,^^30^

When considering the questions regarding places to be physically active, all the places helped to define active behaviour, therefore, showing higher odds of being active. Our data partially agree with previous studies reporting places such as at work,^10^ at home^31^ and at health or fitness centres.^11^^,^^27^ It seems reasonable to continue to promote those places more likely to help achieve PA recommendations as an efficient way of reducing inactive behaviour.

All reasons included in the final model helped to detect active individuals, but with small beta values, indicating that none were particularly indispensable. Factors showing higher beta values were not usually included in other previous studies, such as selecting another reason for being active, developing new skills, and to better integrate into society. Additionally, other factors with lower beta values but coinciding with the literature also were included, such as improving health or physical appearance^7^^,^^32^ and for having fun.^6^^,^^33^ Several other variables were eliminated for the final model, showing that in the presence of particular reasons, other are redundant (e.g. for the spirit of the competition, important in a previous report^7^) or might ask for the same information (e.g. improve health vs. controlling weight).

In regard to membership, being a member of a healthy/fitness facility, sport club or social–cultural club increases the odds of being active.^13^ The reasoning for this, aside from the objective and direct effects on active behaviour, may be that there are other indirect impacts on behaviour, such as interpersonal influences, social support, modelling and expectations, helping to progress in an active lifestyle.^34^

When considering the extent of agreement or disagreement with some statements, from the three questions, only two influenced the final model. Totally agreeing with the statement regarding the extent of opportunities to be physically active in an area vs. totally disagreeing increased the odds of being an active individual, which is in agreement with the literature.^14^^,^^15^^,^^35^ As well, and considering the extent of sport providers, totally agree, tend to agree and tend to disagree vs. totally disagree increases the odds of being an inactive person. Thus, it is plausible that being physically inactive means a lowered understanding of the opportunities available, overestimating the real extent of the PA and sport provision. Contrary to our data, a previous study reported just the opposite.^14^ Finally, and consistently with the literature, the perception of local authorities not doing enough was not included in the final model, showing a general lack of perceived importance.^4^^,^^14^^,^^36^

With regard to the barriers, there was a general pattern of showing increased odds of being inactive.^6^ In line with previous research, the variables with higher odds included having a disability or an illness,^11^ lacking motivation or interest^7^^,^^11^^,^^37^ and being afraid of injuries.^37^ Interestingly, while not having friends to do sport with is understudied in the literature, other variables previously reported were eliminated from our final model, such as not having enough time^11^^,^^37^ or the cost of participating in an active lifestyle.^6^ Additionally, other important barriers are not included in the Eurobarometer, such as lacking energy or reported tiredness^6^^,^^11^^,^^37^ or not liking PA.^11^^,^^37^ In contrast to previous reports, not having infrastructure close-by increased the odds of being physically active.^10^ Considering all barriers altogether, some have a greater effect on being inactive, therefore moving other lesser barriers to a secondary role, while others were eliminated from the final model (i.e. explaining the same variance). This then highlights the first-magnitude barriers seem as the prudent first-step approach to public health interventions aimed at addressing PIA.

It is worth noting that when some questions belong to different contexts, they may overlap around the same content. For example, exercising in fitness centres, being a facility member and perceiving local opportunities ask for the same type of information but from different perspectives. In this way, most of the questions helped to explain active behaviour but not why people remain inactive. Because of this limited capacity for understanding inactive behaviour, other questions should be included in the future Eurobarometers, or new surveys should be designed to support the PA promotion agenda. New surveys should help to develop the key objectives regarding reducing PIA proposed by the World Health Organization.^8^ In this document, the development of policies and monitoring to improve PA implementation was pointed out as a priority to support research, including the evaluation of interventions.^8^ Nevertheless, analyses of programme evaluation in the European Union are scarce, and PIA has not decreased over recent years.^5^ Due to this limited success, the ‘Global Action Plan’ of 2018 attempts to tackle PIA from a broader, more ecological approach to the problem.^8^

Within this perspective, different kinds of questions should be included in future surveys to better understand and intervene more effectively in inactive behaviour. The social situations and stimulus tied to specific reference groups (i.e. family, friends, co-workers), such as social support^14^ and social norms,^32^ are essential to consider. Additionally, exploring with whom an individual is active (i.e. alone or with other people) and if the social environment is physically active is also relevant.^38^ It would be interesting to fully explore active transport,^9^ PA at the workplace,^39^ urban environment,^23^ the influence of weather and seasonality^9^ and several psychosocial variables such as self-identity, intentions, habits, self-monitoring or planning, when also considering previous behaviour.^40^

Our study should consider the following limitations. Firstly, the use of perceived measures based on surveys in a cross-sectional design means that the direction of causality cannot be addressed. Secondly, the influence of perceptions may reflect more the background of the individuals (i.e. age, gender, or physical activity levels) than the actual environment differences.^15^ Lastly, the literature quantified PA levels with different methods and questionnaires, potentially reducing the comparability between then.

## Funding

This article arises from the mobility programme ‘On the Move’ granted to X.M. by the Society of Spanish Researchers in the UK.

*Conflicts of interest*: None declared.

Key points

- The model reported a limited ability to detect modifiable factors affecting physical inactivity, identifying a minimal percentage of inactive individuals correctly.
- The variables asserting the most influence on the model for identifying inactive individuals were having a disability or an illness, not having friends to do sport with, lacking motivation or interest, being afraid of the risk of an injury, and totally agreeing, tend to agree and tend to disagree regarding the extent of local sports clubs and other local providers offering enough opportunities to be physically active vs. totally disagreeing.
- The model was shown to be best designed for active individuals, which is a limitation from a public health perspective.
- New questions should be included in future Eurobarometers, or new surveys should be designed to support the physical activity promotion agenda better.

## Data Availability

The data underlying this article (Special Eurobarometer 412, March 2014) is owned by the European Commission and are available online at: https://dbk.gesis.org/dbksearch/sdesc2.asp?no=5877&search=Physical%20fitness%20and%20exercise&search2=&field=all&field2=&DB=e&tab=0&notabs=&nf=1&af=&ll=10.
